# The Association of Types of Training and Practice Settings with Doctors’ Empathy and Patient Enablement among Patients with Chronic Illness in Hong Kong

**DOI:** 10.1371/journal.pone.0144492

**Published:** 2015-12-14

**Authors:** Frances S. K. Yu, Benjamin H. K. Yip, Kenny Kung, Colman S. C. Fung, Carmen K. M. Wong, Augustine T. Lam, Stewart W. Mercer, Samuel Y. S. Wong

**Affiliations:** 1 Department of Family Medicine, New Territories East Cluster, Hospital Authority, Hong Kong SAR, The People's Republic of China; 2 School of Public Health and Primary Care, The Chinese University of Hong Kong, Hong Kong SAR, The People's Republic of China; 3 Institute of Health and Wellbeing, University of Glasgow, Glasgow, United Kingdom; 4 Department of Family Medicine and Primary Care, The University of Hong Kong, Hong Kong SAR, The People's Republic of China; Sickle Cell Unit, JAMAICA

## Abstract

**Background:**

The increase in non-communicable disease (NCD) is becoming a global health problem and there is an increasing need for primary care doctors to look after these patients although whether family doctors are adequately trained and prepared is unknown.

**Objective:**

This study aimed to determine if doctors with family medicine (FM) training are associated with enhanced empathy in consultation and enablement for patients with chronic illness as compared to doctors with internal medicine training or without any postgraduate training in different clinic settings.

**Methods:**

This was a cross-sectional questionnaire survey using the validated Chinese version of the Consultation and Relational Empathy (CARE) Measure as well as Patient Enablement Instrument (PEI) for evaluation of quality and outcome of care. 14 doctors from hospital specialist clinics (7 with family medicine training, and 7 with internal medicine training) and 13 doctors from primary care clinics (7 with family medicine training, and 6 without specialist training) were recruited. In total, they consulted 823 patients with chronic illness. The CARE Measure and PEI scores were compared amongst doctors in these clinics with different training background: family medicine training, internal medicine training and those without specialist training. Generalized estimation equation (GEE) was used to account for cluster effects of patients nested with doctors.

**Results:**

Within similar clinic settings, FM trained doctors had higher CARE score than doctors with no FM training. In hospital clinics, the difference of the mean CARE score for doctors who had family medicine training (39.2, SD = 7.04) and internal medicine training (35.5, SD = 8.92) was statistically significant after adjusting for consultation time and gender of the patient. In the community care clinics, the mean CARE score for doctors with family medicine training and those without specialist training were 32.1 (SD = 7.95) and 29.2 (SD = 7.43) respectively, but the difference was not found to be significant. For PEI, patients receiving care from doctors in the hospital clinics scored significantly higher than those in the community clinics, but there was no significant difference in PEI between patients receiving care from doctors with different training backgrounds within similar clinic setting.

**Conclusion:**

Family medicine training was associated with higher patient perceived empathy for chronic illness patients in the hospital clinics. Patient enablement appeared to be associated with clinic settings but not doctors’ training background. Training in family medicine and a clinic environment that enables more patient doctor time might help in enhancing doctors’ empathy and enablement for chronic illness patients.

## Background

With the rising prevalence and burden of non-communicable diseases worldwide, primary care was advocated by the WHO (World Health Organization) to address the psychosocial needs of chronic illnesses patients[1], as it is cost effective[2] and people-centered[3]. An Australian study[4] revealed that primary care was also being advocated by patients with chronic illness, who consistently demanded the “right GP” to provide “whole person care” to meet their psychosocial needs on top of their physical conditions.

In Hong Kong healthcare is provided by both private and public sectors. Majority of chronic illness patients (almost 80%) are being taken care of in the public sectors in either community general outpatient clinics (GOPCs) or hospital specialist outpatient clinics (SOPCs).[5] Hypertension was the commonest chronic illness (39.0%), followed by diabetes mellitus (17.8%) and hypercholesterolemia (13.8%). Community general outpatient clinics are the first point of contact for these patients. In fact chronic illness accounts for almost half of all the problems encountered in these public primary care clinics and the volume of consultation is increasing as the population ages.[6] Starting from 2009, the government launched a series of pilot projects to enhance its primary care services in the management of chronic diseases to provide better support for these patients through multi-disciplinary and cross-sector collaboration in their care. For more advanced conditions, patients with chronic illness would be referred to hospital clinics for management, usually to hospital family medicine specialist clinics or medical specialist clinics.

Family medicine specialist clinic in the hospital is a relatively new model of care, which serves as the interface between primary and secondary care. It serves chronic illness patients who need more advanced treatment regimens or investigations. There is enhanced access to multiple specialized investigation tools as supported by medical specialists, including cardiac investigations e.g. holter, treadmill exercise tolerance test, ambulatory blood pressure monitoring; and endoscopic investigations. There is also access to a wider scope of radiological investigations and medications as compared to the community general outpatient clinics. Patients who need further management by internal medicine specialist would be referred to hospital medical specialist clinics. Besides difference in accessibility to investigations and treatment, the consultation time in community clinics and hospital clinics also differ. The consultation time in the community clinics are comparatively shorter as doctors there need to see about 10 patients per hour, while doctors in hospital clinics need to see about 5–7 patients per hour. The majority of doctors in the community clinics have family medicine training and some have no formal family medicine training. In the hospital family medicine and medical specialist outpatient clinics, doctors have family medicine and internal medicine training respectively.

Because of these differences between community and hospital clinics, some patients prefer to have their chronic conditions seen in hospital clinics than in the community clinics for the perceived better equipments, record keeping and doctors’ qualifications.[7] Another barrier identified amongst our local chronic illness patients against the adoption of a “family doctor” model for the care of their illness is their concern about the quality of care by family doctors, who, in their perception, might not have been adequately trained or skilled to manage chronic disease.[8]

Family medicine (FM) training has been advocated to assure and enhance the quality of primary care in Hong Kong. Indeed, family medicine has a proven role in enhancing patients’ care, as its attributes, namely continuity of care, patient centeredness and coordination of care were shown to be associated with improvement in patients’ health, satisfaction and cost of care.[9] Furthermore, residency training in family medicine was shown to improve quality of care in Canada and Thailand.[10–12] According to a local study in 2011, [13] the level of doctors’ training in family medicine correlated positively with patient perceived empathy in primary care patients. However, the effect of family medicine training on perceived empathy for chronic illness patients as compared to other specialist training was not known.

Empathy is important in quality care of patients as a basic component of rapport building in the therapeutic relationship and is essential to patient-centered care (Mercer et al. 2011). It is associated with actual improvement in health outcomes. Diabetes patients of physicians with higher empathy scores were significantly more likely to have good control of HbA1c and LDL.[14] Patients of physicians with higher empathy score had the shortest cold duration and greater change of interleukin-8 and neutrophil level.[15] Doctor’s empathy was also found to increase patient’s enablement,[16] which in turn resulted in greater compliance with treatment and improved health outcomes.[17] Patient enablement refers to the ability of patient to understand and cope with their illness,[18,19] and thus is an important goal of consultation for chronic illness patients.

Our study aimed to explore the association between family medicine training and patients’ perceived empathy and enablement by doctors for chronic illness patients, as compared to that of other specialist training and those without any postgraduate training. We hypothesized that doctors with family medicine training will exhibit higher scores from the CARE Measure than those without or with non-family medicine specialist training. We also want to explore if these scores might be influenced by the different types of clinic settings for the care of these patients, namely hospital and community clinics.

## Method

A cross-sectional study using a questionnaire which included the validated Chinese CARE Measure and Chinese PEI was conducted between Jul 2011 and Jul 2012 in three community clinics (General Outpatient Clinics, GOPCs) and two hospital clinics (Specialist Outpatient Clinics, SOPCs) in the public sector as majority of chronic illness patients in Hong Kong are being followed up in these types of clinics. The two hospital clinics (SOPCs) included the family medicine (FM) specialist clinic and the medical (Med) specialist clinic in a regional public hospital in Hong Kong.

### Settings

We invited doctors based on the 4 sub-groups according to doctors’ training and practice settings as shown in **Table 1**. 15 doctors from the three community general outpatient clinics were invited and two refused. 7 doctors from the family medicine specialist clinic were invited and agreed to participate in the study. For the medical specialist outpatient clinic, 12 doctors were invited and 7 agreed to participate. A total of twenty-seven doctors agreed to take part in the study. Confidentiality was assured to the doctors (their names were not known to the research staff and instead, each doctor was given a number).

**Table 1:** Training background of participating doctors in different clinics.

	Community Clinics		Hospital Clinics	
	GOPCs (General Outpatient Clinics)		SOPCs (Specialist Outpatient Clinics)	
**Training background**	(6 non-trainee)	(7 FM trained dr)	(7 FM trained dr)	(7 Internal Medicine trained dr)
**Level of training**	No specialist training	2 higher trainees; 5 specialist	5 higher trainees; 2 specialist	2 higher trainees; 5 specialist
**Postgraduate experiences(yr)**	15–39	9–10	8–18	9–20
**Mean age**	49	33	34	36
**Gender (M:F)**	4:2	5:2	3:4	2:5

The recruitment of patients and completion of questionnaires were assisted by 11 student helpers. They have received standard training in how to recruit patients and conduct the questionnaire interview if needed. The list of available consultation sessions of each doctor within a three-month period were input into an excel table, to which a list of random numbers were generated by the computer. Consultation sessions were selected accordingly. During the selected consultation sessions of the doctors under our study, student helpers or research assistants would also record the duration of consultation for every patient and invited him/her immediately after the consultation for taking part in the study with informed written consent. Student helpers or research assistants conducted the questionnaires verbally if patients were illiterate or have difficulty in filling in the questionnaires themselves. Patients were excluded from the study if their consultations were not for the follow up of chronic illness. They were also excluded if they were below 18 years of age or were mentally incapacitated to complete the survey. There were no incentives received by patients for participating in the study. Written and verbal information were given to patients about the anonymous nature of the questionnaires and confidentiality of all the information they provided. After completion, questionnaires were then placed in a sealed envelope by the student helpers for collection.

### Outcome Measures

The primary outcome measures were the Consultation and Relational Empathy (CARE) Measure and the PEI. The CARE Measure is a patient-rated experience measure developed in the United Kingdom by Mercer et al. (2004), and has undergone extensive validation.[20–26] The Chinese version of the CARE Measure has been locally validated and was found to be able to reliably differentiate doctors’ patient-rated empathy.[13,27] The ten questions in the CARE Measure were rated on a 5-item response scale from ‘poor’ to ‘excellent’ by patients in response to the question ‘How was the doctor at?’ (e.g. item 1: making you feel at ease’) with a score of 1 for ‘poor’ and 5 for ‘excellent’. The total score was calculated by adding up the ten item scores (and can range from 10 to 50). If there were missing values or ‘not applicable’ item in the response, we re-calculated the total score by calculating the average item score and then multiplying that by 10. Mercer et al. (2011) found that this method of dealing with missing or ‘not applicable’ responses was shown to give similar total scores compared with other approaches such as excluding questionnaires with any missing or not applicable’ and had the advantage of maximizing sample size. [13]

The PEI (Patient Enablement Instrument) was developed by Howie *et al* in the United Kingdom[28], to assess the enablement of patients after a consultation. It has six items which measures the patient’s ability to cope with and understand his/her illness. The Chinese version has been validated locally and was shown to have good validity and reliability.[29] There were six questions in PEI and each question had four response options: “much better/much more”, “better/more”, “same or less” or “not applicable”. The first three responses were scored 2, 1 and 0, respectively, giving a total PEI score ranging from 0 to 12. The calculation of the total PEI score was similar to that of CARE by obtaining the mean of the scores of the applicable items and then multiplied it by six. Cases that had more than three ‘not applicable’ items were excluded.

Besides CARE Measure and PEI, our questionnaires also included information on the number of chronic illnesses being followed up during the consultation and basic demographic information of the patients.

### Sample Size Calculation

Based on the local study by Mercer et al. (2011), the CARE Measure requires 30 patients per doctor in order for the findings to reliably differentiate the performance of respective doctors.[13] We aimed to recruit twenty eight doctors, seven from each of the four groups of doctor. With a total of 840 patients (~30 patients/doctor) and an alpha level equal 0.05, this study has 0.85 power to detect Cohen-d equal or more than 0.2 (i.e., small effect size), after adjusting the design effect by assuming the intraclass correlation is equal 0.1.

### Data Analysis

Mean differences between the four groups of doctors were compared by one-way ANOVA, and the Chi-squared test for differences of proportions. The association between the outcome measures and potential confounders (socio-demographic characteristics, doctor-level factors, clinic visit characteristics) were evaluated by fitting multiple linear regressions. Likelihood ratio test was used to assess the improvements in nested linear models, and Akaike information criteria (AIC) for non-nested models. We used generalized estimation equation (GEE) to account for cluster effects of patients nested with doctors.

### Ethical issue

Ethical approval was obtained from the Joint CUHK-NTEC (Chinese University of Hong Kong–New Territories East Cluster) Clinical Research Ethics Committee.

## Results

### Patient characteristics

Eight hundred and twenty-three patients participated in the study, with an average response rate of 78% (range 62% to 87% per doctor). The number of patients participating per doctor ranged from 30 to 32.

**Table 2**shows the characteristics of the participating patients for the four groups of doctors. There was a slight predominance of elderly patients in the community clinics (GOPCs) among the non-trainee doctor group. In this group, the education level and household income of patients were comparatively lower than those of the other three groups. Household income was highest in patients of the medical trained doctor group in hospital clinics (SOPC).

**Table 2:** Demographic data of participating patients.

*Clinic*	*Community Clinics (GOPCs)*				*Hospital Clinics (SOPCs)*				*P*
*Dr’s training*	(non-trainee)		FM Trained Dr		Med Trained Dr		FM trained Dr		(X^2^)
	n	(%)	n	(%)	n	(%)	n	(%)	
*Age Group*									
*< 45*	5	(2.8%)	11	(5.2%)	42	(20.2%)	33	(14.7%)	P<0.001
*45–64*	71	(39.9%)	111	(52.9%)	87	(41.8%)	99	(44.2%)	(X^2^ = 49.62)
*65+*	102	(57.3%)	88	(41.9%)	79	(38.0%)	92	(41.1%)	
*Gender*									P = 0.078
*Male*	80	(44.7%)	90	(42.7%)	84	(40.6%)	117	(52.2%)	(X^2^ = 6.827)
*Female*	99	(55.3%)	121	(57.3%)	123	(59.4%)	107	(47.8%)	
*Marital status*									P = 0.042
*single*	10	5.6%	12	5.7%	27	13.1%	18	8.5%	(X^2^ = 17.473)
*married*	153	85.5%	165	78.9%	146	70.9%	164	77.4%	
*Separated / Divorced*	4	2.2%	9	4.3%	6	2.9%	5	2.4%	
*widowed*	12	6.7%	23	11.0%	27	13.1%	25	11.8%	
*Education level*									P<0.001
*Primary or below*	98	54.8%	93	44.5%	82	39.6%	91	42.9%	(X^2^ = 37.682)
*Secondary level*	76	42.5%	100	47.9%	83	40.1%	87	41.0%	
*Tertiary or above*	5	2.9%	16	7.7%	42	20.3%	34	16.1%	
*Household income / month*									P<0.001
*≤10000*	98	63.2%	76	46.9%	65	35.1%	78	47.3%	(X^2^ = 36.159)
*10001–40000*	53	34.2%	75	46.3%	96	51.9%	80	48.5%	
*≥40001*	4	2.6%	11	6.8%	24	13.0%	7	4.2%	

### Consultation Duration

The mean duration of consultation differed between the four groups significantly as shown in **Table 3**(P<0.01, χ2 = 179.92), which were 4.5, 5.1, 10.5 and 9.7 minutes in GOPC non-trainee doctor, GOPC FM doctor, SOPC medical doctor and SOPC FM doctor respectively.

**Table 3:** Consultation Duration.

*Clinics*	*Community*		*Hospital*	
	*General Outpatient Clinics (GOPCs)*		*Specialist Outpatient Clinics (SOPCs)*	
*Dr’s training*	(Non-trainee) Dr	FM Trained Dr	Med Trained Dr	FM trained Dr
***Mean Consultation time in minutes (SD)***	4.54 (2.41)	5.076 (2.73)	10.45 (8.56)	9.72 (5.14)
	P = 0.772 between GOPC groups		P = 0.106 between SOPC groups	
	p = <0.001* by ANOVA (between GOPC and SOPC groups)			

### CARE Scores

**Table 4**shows the CARE scores and **Table 5**shows the GEE estimated coefficients of the four group of doctors. Community Clinic (GOPC) doctors without FM training was the reference group and had an estimated mean CARE score of 29.11 (SE = 1.29). Community Clinic FM trained doctor has a higher CARE scores than doctors without training, but the difference was not significant (p = 0.06) (**Table 5**). FM trained doctor from the hospital clinic had significantly higher CARE score (39.04) than those of other doctors, including hospital clinic doctors with non-FM training (3.48 score difference, p-value = 0.04) (**Table 5**). From linear contrast comparisons, hospital clinic doctors had significantly better CARE score than community clinic doctors, irrespective of the doctor training background.

**Table 4:** CARE and PEI Scores.

*Clinic*	*Community Clinics (GOPCs)*		*Hospital Clinics (SOPCs)*	
*Dr’s training*	(non-trainee)Dr	FM Trained Dr	Med trained Dr	FM Trained Dr
*Score*	Mean (SD)	Mean (SD)	Mean (SD)	Mean (SD)
***CARE*** ^***1***^	29.17(7.43)	32.08(7.95)	35.47(8.92)	39.17(7.04)
***PEI*** ^***2***^	3.76(2.43)	3.72(2.57)	4.72(2.91)	5.11(2.88)

^1^ Significant difference in mean CARE score between doctor groups by ANOVA test (P value < 0.01*).

^2^ Significance difference in PEI score between SOPC and GOPC doctor groups by ANOVA test (P <0.05*), but no significance difference between doctor groups within similar clinic practice (either within GOPCs or SOPCs)

**Table 5:** Factors associated with CARE Measure core–unadjusted and adjusted GEE estimates.

	Unadjusted GEE estimates			Adjusted GEE estimates		
Variable	Estimate	SE	*p*-value	Estimate	SE	*p*-value
Intercept*	29.11	1.29	<0.0001	28.88	1.26	<0.0001
GOPCs FM trained doctor	2.88	1.55	0.0640	2.82	1.51	0.0616
SOPCs Med trained doctor	6.44	1.86	0.0005	5.40	1.88	0.0040
SOPCs FM trained doctor	9.93	1.74	<0.0001	8.86	1.64	<0.0001
Male patient				1.72	0.56	0.0021
Consultation time*				0.19	0.06	0.0012

*Mean (7.44 minutes/consultation) standardized time.

As the patients’ demographics and consultation characteristics varied between the four doctor groups (Table 2 and Table 3), we further analyzed the association of type of doctors after adjustment of the potential confounders. Only patients’ gender and consultation time were significantly associated with CARE score (**Table 5**). Male tended to give 1.72 higher CARE score than female and longer consultation time was positive associated with better CARE score (0.19 score per minute increase) (**Table 5**). After adjustment of these factors, the ranking of CARE scores between types of doctors remained the same, but only Hospital Clinic (SOPC) FM trained doctors had a significantly higher CARE score than Community Clinic (GOPC) non-FM trained doctors (8.86 higher score) (**Table 5**). Linear contrast comparisons revealed that hospital clinic FM trained doctors had a significantly better CARE score than hospital clinic medical trained doctors (3.46 higher score, p-value = 0.0417) and community clinic FM trained doctors (6.05 higher score, p-value<0.0001) (**Table 5**).

### PEI

Patients receiving care from hospital clinic (SOPC) doctors had significantly higher PEI scores than those under the care of community clinic (GOPC) doctors without training (**Tables 4 & 6**). However, within same clinic setting comparisons, i.e within hospital clinics or within community clinics, there is no significance difference in PEI score of patients seen by FM trained doctors or by doctors without FM training **(Table 4)**. The results and conclusion were similar after the adjustment of patients’ gender, which was the only associated demographic variable with PEI **(Table 6)**. PEI was positively correlated with CARE score (r = 0.41, p < 0.001).

**Table 6:** Factors associated with PEI measure core—unadjusted and adjusted GEE estimates.

	Unadjusted GEE estimates			Adjusted GEE estimates		
Variable	Estimate	SE	*p*-value	Estimate	SE	*p*-value
Intercept	3.77	0.29	<0.0001	3.61	0.31	<0.0001
GOPCs FM trained doctor	-0.06	035	0.8633	-0.05	0.34	0.8782
SOPCs Med trained doctor	0.97	0.36	0.0063	0.99	0.35	0.0054
SOPCs FM trained doctor	1.37	0.35	0.0001	1.34	0.35	0.0001
Male patient				0.35	0.26	0.1783

## Discussion

### Family Medicine (FM) training and CARE

In a previous local study on primary care patients,[13] doctors’ empathy as assessed by the CARE Measure was shown to correlate positively with level of family medicine training. The primary aim of our study is to determine if family medicine training is associated with higher perceived doctors’ empathy in chronic illness patients as compared to doctors with other specialist training. We achieved this by collecting data from three public community clinics (GOPCs) and two public hospital clinics (SOPCs), as majority of chronic illness patients are being followed up in these types of clinics.

In our study, FM trained doctors were shown to have a higher CARE score than those with non-FM training in the hospital clinic setting. However, FM trained doctors in the community clinics had a lower CARE score than those FM trained doctors in the hospital clinic, despite having similar training background and years of experience. And in the community clinics, FM trained doctor have a higher CARE score than those without the training, but the difference is not statistically significant (**Table 5**).

This might be related to the much shorter consultation duration (4.5–5.1 minutes) in the community clinics as compared to that (9.3–10.5 minutes) in the hospital clinics, which could have been associated with the lower CARE scores in both groups of doctors in the community clinics than those in the hospital clinics, irrespective of their training background. In fact, consultation duration was shown to be a significant positive correlating factor of CARE Measure in our study, and this was also shown by other local studies[13,27] and UK studies. [22–24] The association of consultation length with CARE score might be related to the effect of time constraint on doctors’ behavior, prohibiting their full potential in interacting with patients. Zantinge et al[30] has reported that general practitioners with a subjective experience of a lack of time are less patient-centered. With the limitation of consultation duration, a larger sample size might be required to detect a significant difference in CARE measures in future studies amongst doctors with different training background in the community clinics.

The longer duration of consultation in the hospital clinics might be related to the higher complexity or severity of illness which doctors need to explore into and handle, as compared to those in the community clinics. Whether the increased need for patient–doctor communication in managing more complex chronic illness might be a factor associated with higher CARE score in doctors in the hospital clinics is not known, as current literature has only shown that the number of chronic illness within an individual has no effect on CARE Measure,[13] but there is little literature that explores the effect of severity of illness on CARE measure. Overall, family medicine training was positively associated with CARE measure after adjusting for potential confounders including consultation time and patient’s gender, which confirms the findings from our previous study. [13]

### Family Medicine (FM) training and PEI

Our study showed that PEI was higher in the hospital clinics (SOPCs) than in the community clinics (GOPCs) irrespective of the training background of the doctors. One postulated factor was the higher CARE score of doctors in hospital clinics, as CARE Measure was found to be a significant correlating factor with PEI in our study. This positive correlation between CARE and PEI has also been well proven in local[27] and other studies.[19,26,31–33] Another factor for the higher PEI in hospital clinics maybe owing to the higher patient’s confidence in these clinics as revealed by a local study[7] and a Korean study[34], in which patients prefer to entrust the care of their illness to hospitals for the perceived higher quality of doctors and care with better facilities and access to investigations. The quality of clinic services and facilities might influence patient’s confidence towards their doctors’ management of their illness and thus their enablement by the doctors. A higher PEI was found in male patients, which was also shown to be a positive correlating factor with PEI in a UK study on general practice[35]. Our study did not find any correlation of PEI with consultation time, which was also found not to be a significant correlating factor in previous studies.[31,36]

### Strengths and weakness

Our study has been able to explore the locally validated CARE Measure and PEI score of doctors with different training backgrounds in both community and hospital clinics specifically for patients with chronic illness. It was able to make an objective timed record of the duration of consultations for assessment of its correlation with CARE and PEI while the consultation length in previous local studies [13,27] was measured by patients’ own subjective estimation. It has made an analysis of the interactions of different factors with CARE and PEI by the use of GEE modeling to check for potential confounders.

One of the limitations of the study was that the results might not be generalizable as it was conducted in only one regional hospital and district. Also our study did not attempt to explore the association between doctors’ training and actual health outcomes and further work is required on this.

## Conclusions

Family medicine training is associated with higher patient perceived empathy for chronic illness patients in the hospital clinics. In community clinics, there is also a higher CARE score in doctors with family medicine training than in those without training, but the difference is not statically significant. Patient enablement, however, seems more related to the clinic practice settings as enablement was higher in patients being followed up by doctors in hospital clinics, irrespective of doctor training background. Quality of care for chronic illness patients might be enhanced by family medicine training, while a supportive clinic environment might be important for patient enablement.

## Implications for practice and research

Training in family medicine for doctors as well as organization of practice system to allow optimal consultation time may be conducive to quality care for patients with chronic illness. Future studies might involve also private practices as well as assessment of practice organization factors to explore the influence of these factors on quality of care and patient enablement. The association between family medicine training and health outcomes of chronic illness patients may also be explored in future works.
